# Inflammatory Eye Disease and Immunosuppressive Therapy: A Rheumatologist’s Perspective

**DOI:** 10.7759/cureus.116847

**Published:** 2026-09-24

**Authors:** Thanda Aung

**Affiliations:** 1 Rheumatology, University of California Los Angeles, David Geffen School of Medicine, Los Angeles, USA

**Keywords:** episcleritis, immunosuppression, inflammatory eye disease, methotrexate, mycophenolate mofetil, orbital inflammation, peripheral ulcerative keratitis, rheumatology, scleritis, uveitis

## Abstract

Inflammatory eye disease, including uveitis, scleritis, episcleritis, peripheral ulcerative keratitis (PUK), and orbital inflammation, is among the most consequential extra-articular manifestations encountered by rheumatologists, both because ocular inflammation can cause irreversible vision loss within weeks and because it frequently signals an underlying systemic rheumatic disease. Ophthalmologists establish the anatomic diagnosis and assess the severity of intraocular inflammation; rheumatologists contribute systemic disease evaluation, selection of immunomodulatory therapy, and long-term toxicity monitoring. Neither specialty can optimally manage these patients alone.

Systemic corticosteroids remain the fastest means of controlling acute ocular inflammation but are not a viable long-term strategy given their cumulative ocular and systemic toxicity. Methotrexate and mycophenolate mofetil are the best-supported conventional steroid-sparing agents, with comparable overall efficacy in randomized comparison, although subgroup data suggest that methotrexate may be preferred for posterior/panuveitis and mycophenolate for intermediate uveitis. Among biologics, adalimumab has the strongest randomized evidence for noninfectious intermediate, posterior, and panuveitis; infliximab and rituximab occupy important but more circumscribed, largely observational roles, particularly when ocular disease is associated with Behçet disease or antineutrophil cytoplasmic antibody (ANCA)-associated vasculitis. Janus kinase (JAK) inhibitors are an emerging option for refractory disease, but the supporting evidence remains predominantly observational.

This review synthesizes current evidence from recent guidelines, consensus recommendations, clinical trials, systematic reviews, and observational studies into a practical, phenotype-driven framework for the rheumatologist. It addresses which ocular presentations warrant urgent rheumatologic evaluation, which systemic diseases to consider, when to escalate beyond corticosteroids, how to select among conventional and targeted therapies, and how to structure ongoing multidisciplinary collaboration with ophthalmology.

## Introduction and background

The eye is an immune-privileged organ, yet inflammatory eye disease is one of the more common ways systemic immune-mediated disease first presents to a physician. Uveitis, scleritis, episcleritis, peripheral ulcerative keratitis (PUK), and orbital inflammation are distinct clinical entities with different anatomic targets, systemic associations, and levels of urgency, yet they are nonetheless often lumped together as “the eye stuff” in general rheumatology training. That imprecision has consequences: the correct systemic workup, the correct choice of immunosuppressant, and the correct threshold for urgency all depend on first accurately defining the ocular phenotype [1].

For the rheumatologist, inflammatory eye disease is a genuinely bidirectional problem. Ocular inflammation may be the presenting feature of a previously unrecognized systemic disease, most classically acute anterior uveitis preceding a diagnosis of axial spondyloarthritis, or it may develop in a patient with long-established, otherwise well-controlled systemic disease, in which case ocular and systemic activity can be entirely dissociated. A normal erythrocyte sedimentation rate (ESR) or C-reactive protein (CRP) does not mean the eye is quiet, and a quiet eye does not mean the systemic disease is controlled [2,3].

Historically, management relied heavily on prolonged corticosteroid exposure. The therapeutic landscape has since shifted decisively toward early steroid-sparing immunomodulatory therapy (IMT), first with conventional antimetabolites, followed by biologics and, more recently, targeted synthetic agents, a shift formalized in ophthalmology’s Fundamentals of Care for Uveitis (FOCUS) initiative and increasingly echoed in rheumatology-authored guidance [4,5]. This review synthesizes that evidence into a practical framework organized around the questions a rheumatologist actually has to answer at the bedside: what is inflamed, how urgently must it be controlled, what systemic disease might be driving it, and which therapy best serves both the eye and the patient as a whole.

This narrative review was developed using a structured literature search of PubMed/MEDLINE, Embase, and Google Scholar conducted from July 22 through August 22, 2026. Searches combined terms related to inflammatory eye disease, including “uveitis,” “scleritis,” “episcleritis,” “peripheral ulcerative keratitis,” and “orbital inflammation,” with terms related to systemic rheumatic disease and immunomodulatory therapy. English-language human studies were considered, with priority given to clinical practice guidelines, consensus recommendations, randomized controlled trials, systematic reviews and meta-analyses, and relevant observational studies. Reference lists of key reviews, guidelines, and landmark studies were also examined. Studies were selected for relevance to the diagnosis, systemic evaluation, treatment, and multidisciplinary management of inflammatory eye disease from a rheumatologist’s perspective. Evidence was synthesized narratively, with greater weight given to guideline-level and randomized evidence when available. The review focused on practical systemic evaluation and immunomodulatory management of noninfectious inflammatory eye disease encountered in rheumatology practice.

## Review

Classification and clinical spectrum of inflammatory eye disease

Inflammatory eye disease is not one diagnosis but a family of anatomically defined syndromes, each with its own differential and urgency. The Standardization of Uveitis Nomenclature (SUN) Working Group anatomic classification remains the reference framework for uveitis: anterior, intermediate, posterior, or panuveitis, according to which ocular compartment is primarily involved [6]. Outside the uveal tract, scleritis, episcleritis, PUK, and orbital inflammation are classified by the structure involved rather than by depth within the globe [1].

Anterior uveitis is the most common uveitic phenotype and is strongly associated with the spondyloarthritis spectrum - axial spondyloarthritis, psoriatic arthritis, reactive arthritis, and inflammatory bowel disease-associated spondyloarthritis - as well as juvenile idiopathic arthritis (JIA) and sarcoidosis. Intermediate, posterior, and panuveitis are less common but carry a substantially higher risk of permanent visual impairment, since they more often involve the macula, retina, and optic nerve; their differential includes sarcoidosis, Behçet disease, Vogt-Koyanagi-Harada disease, and systemic vasculitis, alongside a broad range of infections that must be excluded before immunosuppression begins [6,7].

Scleritis and episcleritis are distinguished by the depth of tissue involved - scleritis (deep, potentially vision-threatening) versus episcleritis (superficial, not vision-threatening) - and scleritis carries an especially strong pull toward systemic autoimmune disease, particularly in its necrotizing form. A 2025 analysis of over 41,000 scleritis patients in the US TriNetX database found rheumatoid arthritis to be the most common associated systemic disease, present in roughly 10% of the cohort, with prednisone, methylprednisolone, and methotrexate the most commonly prescribed agents [8]. PUK is a destructive, crescent-shaped corneal stromal process at the limbus that frequently coexists with scleritis and, when associated with systemic vasculitis, is a genuine ocular and systemic emergency [9]. Orbital inflammation - involving the lacrimal glands, extraocular muscles, or orbital fat - is less often on the rheumatologist's radar but includes important rheumatic causes such as GPA, sarcoidosis, and IgG4-related disease, alongside thyroid eye disease and lymphoproliferative mimics [1]. These anatomic categories and their principal systemic rheumatic disease associations are summarized in Table 1.

**Table 1:** Anatomic classification of inflammatory eye disease and associated systemic rheumatic diseases This table summarizes the principal systemic associations reported in published rheumatology and ophthalmology literature [1,6-11] HLA-B27: human leukocyte antigen B27; JIA: juvenile idiopathic arthritis; VKH: Vogt-Koyanagi-Harada disease; MS: multiple sclerosis; RA: rheumatoid arthritis; PUK: peripheral ulcerative keratitis; GPA: granulomatosis with polyangiitis; ANCA: antineutrophil cytoplasmic antibody; IgG4: immunoglobulin G4

Anatomic category	Common systemic associations
Anterior uveitis	HLA-B27–associated spondyloarthritis, JIA, sarcoidosis [1,6,7,10,11]
Intermediate/posterior/panuveitis	Sarcoidosis, Behçet disease, VKH disease, multiple sclerosis MS–associated (pars planitis) [1,6,7]
Non-necrotizing scleritis/episcleritis	RA, relapsing polychondritis [1,8,9]
Necrotizing scleritis and PUK	GPA and other ANCA-associated vasculitides, severe active RA [8,9]
Orbital inflammation	GPA, sarcoidosis, IgG4-related disease, thyroid eye disease [1,7]

Why inflammatory eye disease matters to rheumatologists

Uveitis alone accounts for an estimated 10-15% of legal blindness in high-income countries, disproportionately affecting working-age adults, and a substantial share of that burden is preventable with timely diagnosis and treatment [6,10]. Two features make this a rheumatologic as much as an ophthalmologic problem. First, the ocular diagnosis is made and monitored by an ophthalmologist, but the decision to initiate, intensify, or de-escalate systemic immunosuppression increasingly falls to - or should at least involve - a rheumatologist, particularly once therapy extends beyond topical agents. Second, ocular inflammation can inflict permanent structural damage (cystoid macular edema, glaucoma, cataract, corneal perforation) even when the patient has no systemic symptoms and normal inflammatory markers, so the rheumatologist cannot use systemic disease activity as a proxy for ocular disease activity [1,2].

The practical questions a rheumatologist needs answered are not "which drug treats uveitis" but: Which ocular presentations should prompt rheumatologic evaluation? Which systemic diseases should be screened for, and how aggressively? When does the ocular phenotype alone - independent of systemic activity - justify escalating beyond topical therapy? And which conventional or biologic agent best serves both the eye and whatever systemic disease is present, so the patient isn't on two unrelated drug regimens when one coordinated one would do [1,3].

Systemic Rheumatic Diseases Associated With Ocular Inflammation

Non-infectious ocular inflammation occurs across nearly the entire span of rheumatic disease, but the strength and pattern of association varies by ocular phenotype and is worth internalizing as a rough map:

Axial spondyloarthritis, psoriatic arthritis, reactive arthritis, and IBD-associated spondyloarthritis: Recurrent, typically unilateral-alternating acute anterior uveitis; HLA-B27 positivity increases but does not establish the diagnosis, and uveitis may precede recognized joint disease [6,11].

Juvenile idiopathic arthritis: Chronic, often asymptomatic anterior uveitis, particularly in ANA-positive oligoarticular disease; regular screening by age and JIA subtype is standard of care regardless of ocular symptoms [1].

Sarcoidosis: It can present with any uveitic phenotype, often bilateral, and posterior or panuveitis; may occur without other systemic manifestations and can be the presenting feature of the disease [1,6].

Behçet disease: A prototype cause of severe panuveitis with retinal vasculitis; isolated anterior uveitis without eventual posterior involvement should prompt reconsideration of the diagnosis [1].

Rheumatoid arthritis: The single most common systemic association of scleritis and a major cause of PUK, especially in longstanding, seropositive, erosive disease; necrotizing scleritis or PUK in an RA patient signals a shift toward rheumatoid vasculitis and often warrants treatment as aggressive as major-organ vasculitis [8,9].

ANCA-associated vasculitis (GPA especially): Necrotizing scleritis, PUK, retinal vasculitis, and orbital inflammation; ocular disease may be the presenting or the only manifestation of otherwise localized GPA [9].

Relapsing polychondritis, systemic lupus erythematosus, IgA vasculitis, and IgG4-related disease: Less common but recognized associations, particularly with scleritis and orbital inflammation [1].

A structured, phenotype-directed systemic work-up - rather than an indiscriminate "autoimmune panel" - is the efficient way to approach this: HLA-B27 for recurrent anterior uveitis, ANCA and rheumatoid factor/anti-CCP for scleritis and PUK, ANA/anti-SSA/anti-SSB for JIA- and sicca-associated disease, and serum ACE with chest imaging when sarcoidosis is suspected [1,6].

Diagnostic Approach From the Rheumatologist's Perspective

Before any discussion of immunosuppression, non-infectious etiology has to be established as an active diagnostic conclusion, not an assumption made because the patient carries an autoimmune diagnosis. Herpesviruses, syphilis, tuberculosis, and toxoplasmosis are the dominant infectious mimics in high-income settings, and syphilis serology should be checked in essentially every patient with unexplained ocular inflammation given how protean its presentations are [1,6]. Escalating immunosuppression onto an unrecognized infection is one of the more consequential errors possible in this field.

A directed, rather than shotgun, systemic evaluation follows once infection has been reasonably excluded - guided by the ocular phenotype, laterality, granulomatous versus non-granulomatous appearance on slit-lamp exam, and a genuine review of systems (inflammatory back pain, psoriasis, oral/genital ulcers, sicca symptoms, hearing loss, sinonasal disease). Ophthalmic imaging - optical coherence tomography for macular edema, fluorescein and indocyanine green angiography for vasculitis and choroidal disease - supplements but does not substitute for this systemic evaluation [1,7].

When Rheumatologic Evaluation and Systemic Therapy are Indicated

Several clinical situations reliably justify escalation beyond topical or local corticosteroid therapy, largely independent of whether extraocular systemic disease is apparent: vision-threatening posterior segment involvement; recurrent or chronic disease requiring repeated courses of topical steroid; corticosteroid dependence or intolerance; bilateral severe disease; any necrotizing scleritis; PUK; and, separately, any ocular inflammation occurring in the context of a systemic disease that itself requires systemic treatment [4,5]. The FOCUS initiative operationalized this with explicit triggers - inability to taper prednisone below roughly 7.5-10 mg/day within three months, relapse on tapering, steroid intolerance, or posterior/macular involvement - that remain a useful checklist [4].

The treatment goal should be explicit rather than implicit: sustained ocular quiescence with the minimum cumulative corticosteroid exposure required to get there, not simply "quiet eye, whatever it takes." That framing is what should drive the choice and sequencing of steroid-sparing therapy discussed below.

Corticosteroids: necessary for induction, not a long-term strategy

Corticosteroids remain the fastest and most reliable way to control significant ocular inflammation, whether topical (anterior segment disease), periocular or intravitreal (posterior segment), or systemic (oral prednisone or, for severe/vision-threatening disease, pulse intravenous methylprednisolone). Their acute efficacy is not in question. Their long-term use is the problem: cataract, glaucoma, and infection on the ocular side; osteoporosis, diabetes, hypertension, weight gain, cardiovascular risk, adrenal suppression, and infection systemically. Consensus guidance sets a target long-term maintenance dose of prednisone-equivalent ≤7.5 mg/day, and failure to reach that target within roughly three months is itself an indication for steroid-sparing therapy [4,6]. Corticosteroids are best conceptualized as induction or rescue therapy - a bridge to a steroid-sparing regimen - rather than as the default long-term strategy for chronic inflammatory eye disease.

Conventional immunosuppressive therapies

Methotrexate is the most widely used first-line steroid-sparing agent for chronic uveitis and non-necrotizing scleritis, generally dosed at 15-25 mg weekly (oral or subcutaneous) with folic acid supplementation - familiar territory for any rheumatologist. It has particularly good evidence in scleritis: a retrospective cohort reported corticosteroid-sparing success in 92.3% of patients at 12 months, with a systematic review and meta-analysis subsequently reinforcing its efficacy signal in scleritis, though the authors were explicit that the absence of head-to-head randomized comparator trials limits any claim of superiority over steroids or placebo [12,13,14].

Mycophenolate mofetil (typically 2-3 g/day) is the other principal first-line antimetabolite, often preferred in posterior/panuveitis, in patients with methotrexate intolerance, or when a somewhat faster steroid-sparing effect is desired. The landmark FAST trial (First-line Antimetabolites as Steroid-sparing Treatment) directly compared methotrexate and mycophenolate as first-line therapy for noninfectious intermediate, posterior, and panuveitis: at six months neither drug was statistically superior overall, though methotrexate performed somewhat better for posterior/panuveitis and mycophenolate achieved treatment success somewhat faster with a trend toward fewer side effects [15]. The clinically useful takeaway for a rheumatologist is not that one drug is better, but that there is no universally superior conventional agent - the choice should be driven by phenotype, comorbidity, pregnancy planning, and tolerability, exactly as one would choose between conventional DMARDs for arthritis.

Azathioprine remains a reasonable option, particularly in Behçet disease and where pregnancy planning or specific contraindications steer away from methotrexate or mycophenolate; thiopurine methyltransferase or equivalent pharmacogenetic assessment before initiation is prudent given the risk of severe myelosuppression [1].

Calcineurin inhibitors and cyclophosphamide occupy narrower roles. Cyclosporine and tacrolimus can act quickly in posterior uveitis and Behçet disease but are limited long-term by nephrotoxicity and hypertension. Cyclophosphamide retains an important, if now less frequently first-reached-for, role in immediately sight-threatening or organ-threatening disease - most classically, necrotizing scleritis or PUK reflecting active systemic vasculitis - where it is increasingly used alongside or supplanted by rituximab [1].

Biologic and targeted therapies

TNF Inhibitors: The Best-Supported Biologic Class

Adalimumab has by far the strongest randomized evidence of any biologic in noninfectious uveitis and is the only systemic biologic with FDA approval for noninfectious intermediate, posterior, and panuveitis in adults and children ≥2 years. In VISUAL I, patients with active disease treated with adalimumab had a significantly longer median time to treatment failure than placebo (24 vs 13 weeks; hazard ratio 0.50) [16]; VISUAL II, in patients with inactive disease maintained on corticosteroids, showed a similarly significant reduction in risk of treatment failure (hazard ratio 0.57) [17]. In JIA-associated uveitis, the SYCAMORE trial established superior efficacy of adalimumab combined with methotrexate over methotrexate alone [18]. For a rheumatologist, the practical point is that the evidence base for adalimumab is categorically stronger than for any other biologic in this space - it is the natural second-line agent after conventional antimetabolites fail, and, encouragingly, is also a familiar, guideline-anchored drug for many of the systemic diseases (spondyloarthritis, JIA) that co-occur with uveitis.

Infliximab is used off-label, particularly when rapid control of severe or refractory disease is needed - sight-threatening posterior uveitis, retinal vasculitis, or refractory scleritis, especially in Behçet disease. A small, uncontrolled pilot study found all five patients with active anterior scleritis achieved quiescence by week 14 [19]; larger observational cohorts support a similar signal in refractory scleritis, though direct comparative trial data against adalimumab in uveitis are lacking.

A point worth stating plainly because it changes prescribing behavior: etanercept is not interchangeable with monoclonal anti-TNF agents in ocular disease. It has not shown efficacy for uveitis and has been associated with new-onset or paradoxical uveitis flares [1,2,3]. A patient with spondyloarthritis and recurrent uveitis who needs a TNF inhibitor should not simply be switched to whichever anti-TNF agent is most convenient - the ocular phenotype should influence the choice, and this is a concrete example of where ophthalmology-rheumatology collaboration changes the actual prescription written.

IL-6 Inhibition

Interleukin-6 has a well-established role in intraocular vascular permeability and cystoid macular edema. The STOP-Uveitis study found that intravenous tocilizumab significantly improved visual acuity, reduced vitreous haze, and resolved refractory uveitic macular edema, positioning IL-6 blockade as a reasonable option specifically for uveitis complicated by treatment-refractory CME, and - per the Nature Reviews Rheumatology guidance for rheumatologists - as the best-evidenced second-line biologic class after TNF inhibitors [3,20].

B-Cell Depletion: Rituximab

Rituximab's clearest niche is ocular inflammation occurring in the context of B-cell-mediated systemic disease, above all ANCA-associated vasculitis, where it is often chosen because it is already the appropriate systemic therapy rather than because it was selected specifically "for the eye." A systematic review synthesizing individual data from 229 published patients treated with rituximab for refractory noninfectious uveitis or scleritis found it was generally used third-line or later, after multiple prior immunosuppressive agents had failed [21]. Dedicated scleritis series report favorable results: 93.3% clinical improvement with 86.6% reaching a scleritis activity score of zero at six months in one cohort [22], and 83% remission within three months, sustained for an average of 32 months, in another [23]. This is encouraging but remains observational evidence - not randomized trial data - and should be weighed accordingly, with rituximab most compelling when the systemic disease itself already argues for B-cell depletion.

Janus Kinase (JAK) Inhibitors: A Promising but Still Emerging Class

JAK-STAT signaling sits downstream of multiple cytokines relevant to intraocular inflammation - interferons, IL-6, IL-12, IL-17, IL-23 - which is the mechanistic rationale for JAK inhibitors (tofacitinib, baricitinib, upadacitinib) in refractory disease. The evidence, however, is still almost entirely observational. A 2025 systematic review of 22 case reports and series identified 43 patients (64 eyes), with scleritis accounting for roughly 47% of reported conditions and uveitis for roughly 35%, most having failed both conventional DMARDs and biologics before JAK inhibition [24]. A single-center case series of eight patients similarly reported disease control in most patients initiated on tofacitinib or upadacitinib after failing prior therapy, again with a small, uncontrolled design [25]. A dedicated review of JAK-STAT inhibition in noninfectious uveitis reached the same conclusion: promising signal, no randomized controlled trial evidence yet [26].

For the rheumatologist, this places JAK inhibitors squarely as a fourth-line option for disease refractory to conventional DMARDs and at least one biologic - not a first- or second-line choice - and one that carries the same systemic safety considerations (infection, thrombosis, cardiovascular and malignancy risk, laboratory monitoring) that already govern their use in rheumatoid arthritis and other rheumatic disease.

Disease-specific treatment considerations

Necrotizing Scleritis and Peripheral Ulcerative Keratitis

These two entities deserve to be treated as a single high-urgency category. Necrotizing scleritis presents with severe, boring pain and avascular scleral patches and can progress to scleromalacia perforans if untreated; PUK presents as crescentic peripheral corneal thinning with an epithelial defect. Both frequently reflect active systemic vasculitis - most often GPA or severe seropositive RA - and both should be managed as a systemic emergency rather than a local ocular problem: high-dose systemic corticosteroids (often pulse IV methylprednisolone) combined with prompt induction immunosuppression, typically rituximab or cyclophosphamide when GPA-associated disease is confirmed or strongly suspected [8,9]. A critical, easily missed point: topical corticosteroids are relatively contraindicated as monotherapy in active PUK, since they can accelerate corneal melting and perforation [9].

Behçet Disease

Panuveitis with retinal vasculitis is the dominant ocular threat; anti-TNF therapy (infliximab or adalimumab) has emerged as first-line systemic therapy for sight-threatening Behçet uveitis in current practice, reflecting both efficacy data and the practical decline in availability of interferon-alpha [1].

JIA-Associated Uveitis

Often asymptomatic and detected only through scheduled screening; methotrexate remains first-line steroid-sparing therapy, with adalimumab (supported by the SYCAMORE trial [18]) as the standard second-line biologic when methotrexate alone is insufficient.

A practical framework for selecting systemic therapy

Rather than asking "which immunosuppressant is best for uveitis," the more useful clinical question is which therapy best serves this patient's ocular phenotype, systemic disease, comorbidities, and long-term risk simultaneously. Table 2 summarizes a phenotype-directed starting framework across common clinical scenarios; these are starting points, not an algorithm to be applied rigidly - the evidence base is heterogeneous, and treatment should remain phenotype- and patient-driven.

**Table 2:** Practical framework for selecting systemic therapy by clinical scenario in inflammatory eye disease This table is compiled from published clinical trial and cohort data on systemic and biologic therapy selection in inflammatory eye disease [1-4,8,9,12–17,20-26] SpA: spondyloarthritis; TNF: tumor necrosis factor; MTX: methotrexate; MMF: mycophenolate mofetil; PUK: peripheral ulcerative keratitis; GPA: granulomatosis with polyangiitis; DMARD: disease-modifying antirheumatic drug; JAK: Janus kinase

Clinical situation	Reasonable systemic strategy
SpA	Treat the systemic SpA; consider a monoclonal TNF inhibitor (not etanercept) if uveitis is frequent or severe [1–3]
Intermediate/posterior/panuveitis, corticosteroid-dependent	MTX or MMF first-line; escalate to adalimumab if inadequate control [3,4,15–17]
Refractory uveitic macular edema	Consider tocilizumab in selected refractory cases [3,20]
Necrotizing scleritis or PUK, GPA-associated	Treat as systemic vasculitis: pulse corticosteroids plus rituximab or cyclophosphamide [8,9]
Chronic non-necrotizing scleritis	MTX or MMF; biologic (TNF inhibitor or rituximab) for refractory disease [12–14,21–23]
Refractory to conventional DMARD plus ≥ 1 biologic	Consider a JAK inhibitor in selected patients, with full discussion of the limited evidence base and systemic safety monitoring [24–26]

Collaboration between rheumatology and ophthalmology

Ophthalmology and rheumatology monitor genuinely different, complementary dimensions of the same disease. Ophthalmology tracks visual acuity, anterior chamber and vitreous inflammation, retinal and optic nerve findings, macular edema on OCT, and intraocular pressure. Rheumatology tracks systemic disease activity, medication toxicity (marrow, hepatic, renal, infectious, malignancy risk), corticosteroid burden, and the emergence of systemic manifestations over time [1,3]. Neither surveillance stream substitutes for the other, and the recurring failure mode in these patients is one specialty assuming the other's monitoring covers a domain it does not.

The strongest argument for genuinely coordinated care is that it lets a single therapy address both the eye and the systemic disease at once, rather than layering an ocular-specific drug onto an unrelated systemic regimen: adalimumab or another appropriate TNF inhibitor for a patient with spondyloarthritis and recurrent uveitis; methotrexate or a biologic that simultaneously covers RA-associated scleritis and joint disease; rituximab addressing both GPA-associated scleritis/PUK and the underlying vasculitis. This organ-integrated approach, rather than treating the eye and the joints as separate problems assigned to separate specialists, is increasingly what current rheumatology-facing guidance recommends [2,3].

Monitoring, safety, and treatment-related considerations

Baseline evaluation before systemic immunosuppression should be phenotype-directed rather than a blanket panel: CBC, comprehensive metabolic panel, hepatitis B/C and tuberculosis screening before biologics, and disease-specific serologies (ANCA, RF/anti-CCP, ANA/anti-SSA/anti-SSB, HLA-B27, serum ACE) chosen according to the ocular presentation [1,6]. Ongoing monitoring should track both the expected toxicities of the chosen agent (cytopenias and hepatotoxicity with methotrexate; GI intolerance and cytopenias with mycophenolate; infection and reactivation risk with biologics; the expanded infection, thrombosis, cardiovascular, and malignancy surveillance that accompanies JAK inhibitors) and corticosteroid-related toxicity for as long as steroids remain part of the regimen - bone density, glucose, blood pressure, and ophthalmic surveillance for steroid-induced glaucoma and cataract [6].

Current unmet needs and future directions

Despite meaningful progress, several gaps persist. Head-to-head randomized trials comparing JAK inhibitors against established biologics in uveitis and scleritis are essentially absent, so their current use rests on case series and systematic reviews of case-level data rather than controlled evidence [24-26]. Standardized, validated biomarkers to guide individualized drug selection remain elusive. Tapering protocols and criteria for durable drug-free remission are unstandardized across both ophthalmology and rheumatology, producing wide practice variation [3,5]. A recent large-scale review of the immunomodulatory therapy landscape for noninfectious uveitis specifically flagged the absence of reliable remission-inducing strategies and poorly standardized tapering as the field's central unmet needs, alongside emerging interest in AI-assisted, biomarker-informed personalization of therapy [27].

## Conclusions

Inflammatory eye disease sits at a genuine interface between ophthalmology and rheumatology, and treating it well requires more than reaching for an immunosuppressant. It requires establishing exactly what is inflamed, confirming that infection has been excluded, judging how urgently the disease must be controlled, identifying which systemic disease may be driving it, and selecting a therapy that serves the eye, the systemic disease, and the patient’s long-term safety simultaneously. Corticosteroids remain indispensable for rapid induction but should routinely prompt a plan for steroid-sparing therapy rather than prolonged, open-ended use. Methotrexate and mycophenolate mofetil remain the best-supported conventional agents, with broadly comparable efficacy but different practical advantages in specific clinical situations. Adalimumab has the strongest randomized evidence of any biologic for noninfectious intermediate, posterior, and panuveitis; infliximab, tocilizumab, and rituximab occupy narrower but well-defined roles, particularly when the systemic disease itself supports the use of the same drug. JAK inhibitors are a genuinely promising emerging option for refractory disease, but their current evidence base is predominantly observational, and their use should be framed accordingly. The best outcomes in this field are achieved not by an ophthalmologist and a rheumatologist independently treating different organs, but by a coordinated team working together to treat one patient with one interconnected immune-mediated disease.
